# An automated real-time integration and interoperability framework for bioinformatics

**DOI:** 10.1186/s12859-015-0761-3

**Published:** 2015-10-13

**Authors:** Pedro Lopes, José Luís Oliveira

**Affiliations:** DETI/IEETA, Universidade de Aveiro, Campus Universitario de Santiago, Aveiro, 3810-193 Portugal; IEETA, Campus Universitario de Santiago, Aveiro, 3810 – 193 Portugal

**Keywords:** Data integration, Interoperability, Publish/subscribe, Integration-as-a-service, Intelligent ETL, Workflow, Cloud, Service-oriented architecture, Event-driven

## Abstract

**Background:**

In recent years data integration has become an everyday undertaking for life sciences researchers. Aggregating and processing data from disparate sources, whether through specific developed software or via manual processes, is a common task for scientists. However, the scope and usability of the majority of current integration tools fail to deal with the fast growing and highly dynamic nature of biomedical data.

**Results:**

In this work we introduce a reactive and event-driven framework that simplifies real-time data integration and interoperability. This platform facilitates otherwise difficult tasks, such as connecting heterogeneous services, indexing, linking and transferring data from distinct resources, or subscribing to notifications regarding the timeliness of dynamic data. For developers, the framework automates the deployment of integrative and interoperable bioinformatics applications, using atomic data storage for content change detection, and enabling agent-based intelligent extract, transform and load tasks.

**Conclusions:**

This work bridges the gap between the growing number of services, accessing specific data sources or algorithms, and the growing number of users, performing simple integration tasks on a recurring basis, through a streamlined workspace available to researchers and developers alike.

## Background

The scale of information available for life sciences research is growing rapidly, bringing increasing challenges in hardware and software [1, 2]. The value of these raw data can only be proved if adequately exploited by end-users. This reinforces the role of integration and interoperability at several service layers, allowing them to focus on the most relevant data for their research questions [3].

Biomedical data are complex: heterogeneously structured, originating from several different sources, represented through various standards, provided via distinct formats and with meaning changing over time [4, 5]. From next generation sequencing hardware [6] to the growing availability of biomedical sensors, tapping this on-going data stream is an unwieldy mission [7]. Accessing, integrating and publishing data are essential activities for success, common to commercial and scientific research projects [8]. Many life science researchers perform these tasks on a regular basis, whether through the manual collection and curation of data, or the use of specific software [9, 10].

In recent years, data integration and interoperability is focused on three interdependent domains: cloud-based strategies [11, 12], service-oriented architectures [13] and semantic web technologies [14].

Cloud-based approaches are adequate for institutions that want to delegate the solution for computational requirements. Processing high throughput sequencing data or executing intensive analysis algorithms involves a technical layer that is greatly enhanced by using grid- and cloud-based architectures [15]. This removes any technological hardware concern from the researchers' work. In addition, improved availability and ease-of-access, to and from cloud-based resources, further promotes the use of cloud-based strategies. However, for the majority of researchers, their problems are much smaller and focused, and, where the implementation of a full cloud-based stack is relevant, access to these resources is difficult and expensive.

Workflow management tools represent a leap forward for service interoperability in bioinformatics. The ability to create comprehensive workflows eased researchers’ work [16]. Nowadays, connecting multiple services and data sources is a recurring task. Yet, tools such as Yabi [17], Galaxy [18] or Taverna [19] lack automation strategies, essential for real-time features.

The Semantic Web paradigm has been promoted as a perfect fit for the innate complexity of the life sciences. The complex biological data relationships are better expressed through semantic predicates than what is possible in relational models or tabular files [20]. Although applications such as the semantic Diseasecard [21] or architectures like Semantic Automated Discovery and Integration (SADI) [22] already commoditize Semantic Web technologies, this paradigm is not a “one size fits all” solution.

In this work we introduce an open-source framework to streamline data integration and web service interoperability tasks. Our goals are two-fold: enabling the automated real-time, reactive or event-driven analysis of data, and empowering the creation of state-of-the-art applications.

Traditional integration approaches, in use by data warehouses, rely on batch, off-line Extract-Transform-Load (ETL) processes. These are manually triggered on regular intervals of downtime, which can range from weeks to months or even years. However, in the life sciences domain, the demand for fresh data cannot be ignored. Hence, we need to deploy new strategies that are dynamic [23], reactive [24] and event-driven [25, 26]. Thus, today’s platforms must act intelligently, i.e., in real-time and autonomously, to changes detected in integrated environments.

There is untapped potential in the real-time and event-driven integration of data. Researchers want to access the most up-to-date datasets; thus, ensuring that data are synchronized across resources continues to be an on-going challenge. Combining this need with the resources’ heterogeneity and distribution, and we have a major bottleneck for faster scientific progress.

Our approach is based on the creation and deployment of intelligent agents. Agents track data changes on remote data sources, identifying new events based on user-specified conditions. Next, events trigger the processing of actions based on user-specified templates. Agents monitor resources in CSV, JSON, XML or SQL formats, which cover the majority of data sources and services available. Templates handle integration actions, interacting with files, databases, emails or URLs. As a result, there are endless combinations for customisable integration tasks, connecting events detected by the agents with actions configured in templates. Among others, the framework empowers live data integration and heterogeneous many-to-many interoperability. In addition to the mentioned integration scenario, this platform is suitable to several other research problems within and beyond the life sciences.

## Implementation

The framework’s current version includes two components: server and distributed clients, both available to use and download at https://bioinformatics.ua.pt/i2x/.

The server is the main platform component, powering the framework’s core features. The distributed client scripts enable deploying local agents, using an internal packaged library.

The source code is available at GitHub (https://bioinformatics.ua.pt/i2x/docs/install/index), under the MIT free software license.

### Architecture

**Fig. 1:**
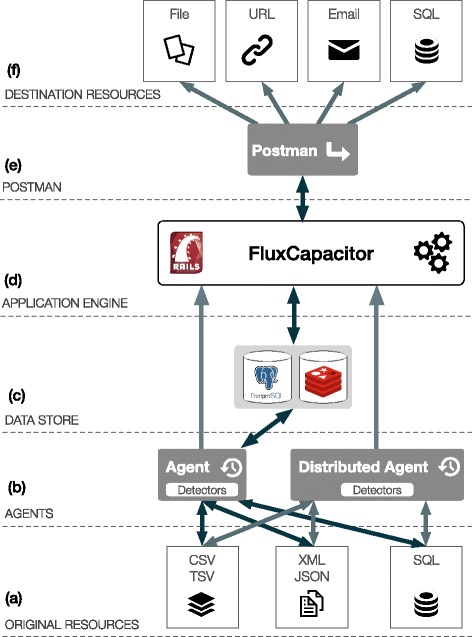
Framework architecture highlighting the different system layers. **a** external Original Resources are accessed for data extraction; **b** local or distributed Agents poll Original Resources; **c** the internal Data store uses a relational database (PostgreSQL or MySQL) to store data and an object cache (Redis) for improved performance; **d** the application engine, is implemented in Ruby, with the Rails framework, and controls the entire application and its API; **e** the Postman applies the data extracted by the Agents to the Templates and executes the final delivery; **f** the external Destination Resources receive the data from the system.

Figure 1 details the framework’s architecture, including its basic components, described next.

**Original resources** refer to the data sources being monitored by the configured agents. In our initial version these endpoints can provide data in SQL, CSV/TSV, XML or JSON format.

**Agents** are intelligent distributed engines tracking resources. Deployed agents form a multiple agent system focused on extracting content for verification in format-specific algorithms. Internally, each agent analyses resources detecting changes in comparison to previous states. Agents are modelled to include the configuration properties required to setup automated real-time content change detection from heterogeneous resources. This includes miscellaneous agent features such as scheduling, endpoints, connection strings, data selectors or caching definitions, among others.

Agents are executed in the main server or in remote locations using the framework’s distributed client scripts. This results in improved security as data exchanges between the original data sources and the server component are reduced, and all transactions are performed via HTTPS.

The agent’s monitoring scheduling is also flexible, as available schedules are not hard-coded. The schedule list is defined in the application settings and can change on distinct server instances. More importantly, whereas the basic *modus operandi* is reactive integration relying on scheduled polling, content changes can also be pushed directly to agents, enabling passive integration.

Within the agents we have **detectors**. These are the algorithms that perform the actual resource tracking. Detection algorithms look for changes in the output content from the origin resources. The detector connects to the data store to analyse, retrieve and compare event metadata obtained by the agents.

In the current implementation, detectors can identify changes in four distinct formats: CSV/TSV files, SQL databases (MySQL or PostgreSQL), XML or JSON data. These formats cover the output of the majority of data sources and web services available. For instance, we can write complex queries that fully explore SQL’s potential to extract data for detection. Moreover, this open architecture makes it easy to build on these methods to expand the detection to extra formats.

Agents can be configured with one or more **Seeds**. Seeds enable the dynamic population of values in the agents’ configuration. Although agents are flexible enough to cover the collection of data from disparate sources, there are scenarios where we need to launch agents for a very large number of targets with a similar configuration (for example, to iterate over a list of identifiers such as genes, proteins or publications). This is where Seeds are used. Seeds’ configuration is identical to agents’ as the framework uses similar selectors to obtain seed values that fill in the gaps in the associated agents’ configuration, enabling the dynamic creation of agents. A sample scenario where seeds can be helpful revolves around the extraction of data from XML content feeds. The outcomes of agents’ monitoring process are **Events**. Each content/state “change” detected by the agent creates a new event. This triggers the execution of actions through delivery templates, enabling a controlled flow of data within distinct resources. Events are atomic and contain the metadata required for finalizing the integration task.

The internal **data store** combines a relational database with a high-performance cache to persist application data. The platform uses the relational database to store all internal data, from agents’ configurations to user details. In addition to these, it stores all the required content verification elements. In addition, the system also features a Redis cache for faster change detection and event generation.

The **FluxCapacitor** is the main application controller, registering and proxying everything. With all components deployed independently, the FluxCapacitor connects all components, acting as as a flow manager and ensuring that all operations are performed smoothly, from event detection to the final delivery. Moreover, FluxCapacitor also enables the framework’s API, empowering the various platform web services.

The **Postman**, as implied by its naming, performs the delivery of each template. That is, it finalises the integration tasks, receiving event data and applying them to the delivery template for execution.

**Destination resources** are the templates’ objects to which the Postman will connect for the final delivery. Delivery **Templates** define the actions executed for new events. The current version contains templates for executing SQL queries, sending emails, calling web services (using URL routes) or managing files (in a private user workspace or in an associated Dropbox account). Like agents, the templates set can be extended with additional plugins to connect custom services or delivery types.

To connect agents and templates we have **Integrations**. That is, integrations define the data origin and destination. Integrations match multiple agents with multiple templates, enabling a many-to-many data distribution.

At last, the **log** engine stores summary information for all actions and flows. Each log entry contains the minimal set of information required to re-enact specific transactions or errors. This includes timestamps, origin/destination and the messages sent. For improved tracking and analysis, this backend uses the Sentry platform.

Agents, templates and integrations’ metadata are stored in the internal database. We devised a flexible and dynamic data model that allows the easy configuration and update of these properties via the platform’s web interface. Further details regarding the internal framework architecture and model are available on the documentation at https://bioinformatics.ua.pt/i2x/docs.

### System workflows

**Fig. 2:**
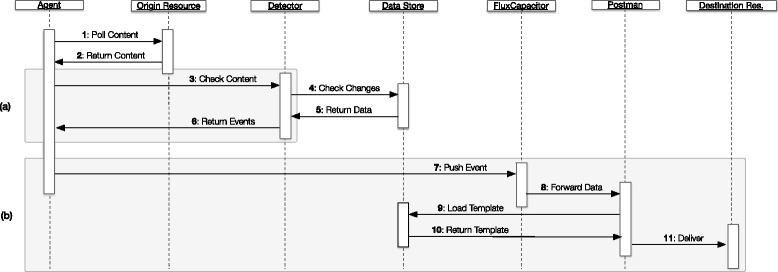
Framework monitoring and integration sequence diagram. In addition to the listed steps, all actions are logged internally for auditing, error tracking and performance analysis. Two alternative pipelines can be executed: **a** distributed agents generate a different sequence from step 3, where FluxCapacitor mediates all interactions; **b** events data can be pushed directly into the platform, generating a new sequence starting at step 7.

Figure 2’s simplified sequence diagram features the framework’s Extract-Transform-Load pipeline, from data source polling to resource delivery. The sequence steps are listed next.

1. Poll Content (*Agent-Origin resource)*: the agent connects to external data sources and loads content.
2. Return Content (*Origin resource-Agent)*: the data source returns the requested content.
3. Check Content (*Agent-Detector)*: the agent sends acquired data to Detector for content change detection.
4. Check Changes (*Detector-Data store)*: the Detector imports the data into the internal Data store and checks if there have been any changes since the last update (new events).
5. Return Data (*Data store-Detector)*: the Data store returns a dataset with the unique new content.
6. Return Events (*Detector-Agent)*: the Detector generates a set of events and returns them to the Agent.
7. Push event (*Agent-FluxCapacitor)*: the Agent pushes event data iteratively (one connection for each event) to the FluxCapacitor
8. Forward data (*Flux Capacitor-Postman)*: the FluxCapacitor sends event data to the Postman for delivery.
9. Load template *Postman-Data store*: the Postman loads the configured templates from the Data store.
10. Return template *Data store-Postman*: the Data store returns matching templates.
11. Deliver *Postman-Destination resource*: the Postman performs the final delivery, transforming the template with the event data, thus concluding the ETL workflow.

There are two more pipelines enabled that imply minimal changes to the framework’s base pipeline: a) when dealing with remote agents, and b) when event data are pushed from external resources. The latter complements the original reactive polling-based approach, which relies on recurring data fetching from the original sources, with a passive push-based strategy, where the original sources must send the new data to an open endpoint in our platform.

For the first alternative (Fig. 2a), there are a couple additional steps on the content detection sequence. The FluxCapacitor mediates the content change detection, acting as a middleware layer between the remote agent and the detection engine. This means that remote agents only interact with the main application controller and API, the FluxCapacitor.

On the second scenario (Fig. 2b), when external providers push events directly, the content detection sub-sequence is ignored. This is where our platform achieves optimal performance. As it is not necessary to fetch data from the original sources, we can skip the change detection algorithm and start the event processing immediately. The internal pipeline starts on step 7 as data are pushed directly from the external resources to the API. In this case, the original data sources are responsible for sending new data to the platform’s API. The drawback of this approach is that it implies implementation changes in the original data sources.

#### Agent distribution

This distributed architecture relies on the ability to deploy intelligent remote agents. Agents can be executed in the server-side, on the same machine as the server component, or at the client-side, where there is direct access to resources.

This feature is relevant when we consider the amount of data available in legacy systems, such as relational databases or CSV files, which are not available through public URLs or services, and also secure private environments. In these scenarios, client agents are configured with the server details, and deployed on the resource location.

A script controls local agent execution, loading all required code through a standalone library. The ETL workflow for distributed agents is similar to what happens in the main server. The major change regards the content change detection engine. Whereas with server-side agents the cache verification occurs within the server codebase, with client-side agents a web service call to the server API performs the verification. Likewise, with new events, the client agent initiates the delivery through another web service call.

#### Dynamic content change detection

To ensure a reliable stream of changed data, this framework relies on a set of content change detection services. As we cannot pre-emptively identify what is new in the original data sources, these services perform basic detection and filtering actions [27], specialised for each integrated data format. This enables the rapid identification of new events. The architecture adopts an atomic data storage approach for content change detection. That is, agents are configured to extract defined data elements from the original sources, and each of these is independently stored, without dependencies to other resources, datasets or agents. This verification process occurs in four steps.

1. A new detector loads agent metadata according to the resource data format. For instance, the CSV detector implementation is different from the SQL database detector one, although both share the same interface.
2. The detector polls the resource for data, which returns the requested dataset. Again, this process is detector-based, requiring format-specific algorithms.
3. The detector validates the retrieved data in an internal cache. The cache acts as the atomic data storage component, storing each data element uniquely.
4. If the retrieved data are not in the cache, i.e., data are fresh, a new event is created, triggering the data push to the main application controller for delivery and storing the new data in the cache. If the data are not new, the integration process stops.

By default, the platform resorts to a solution based on the internal relational database to store events metadata for verification. To improve detection performance, the framework can use a Redis cache instead, making the content change detection process faster and more efficient.

The cache verification process can use two distinct data elements. On the one hand, users can configure the cache property for each agent specifying what variable the change detection algorithm will monitor. This should be set to track unique data properties, namely identifiers. On the other hand, if there are no data elements that can identify integrated data unequivocally, the framework creates and stores an MD5 hash of the data elements’ content. As changing content results in a new hash, we can detect new events without compromising the system performance.

#### Job processing

Scalability issues in automated real-time integration systems are a cause for concern. For example, in modern data processing it is mandatory that information is processed as efficiently as possible. Hence, several challenges surface at the integration and interoperability level. Scalability, performance, processing time or computational requirements are some of the bottlenecks we face. To tackle them, we employ a queue-based approach to control the on-demand execution of monitoring jobs. Since these are the more computationally intensive algorithms, each monitoring task is placed on a homogeneous queue, without priority ordering. During the predetermined scheduling intervals, the framework launches the respective agents.

In parallel with the application server, a queue tracking service is continuously running, dequeuing jobs according to the system’s resources load. For instance, if the system can only execute two tasks simultaneously, and there are four monitoring tasks on the queue, they will be processed two at the time, in order of arrival. Tasks start with the agent monitoring and proceed until the final delivery. Moreover, the job execution daemon is flexible enough to allow distributing the load through multiple processing cores. As such, for computationally intensive tasks we can fully explore multithreading capabilities to optimize the overall system performance.

In spite of being a rather simplistic scaling method, this strategy prevents system overloads without compromising the near real-time solution, i.e., introduced delays are not significant to the application workflow.

#### Extracting, transforming and loading data

At a basic level, this proposal introduces an intelligent ETL proxy. The framework simplifies the process of extracting, transforming and loading data from distributed sources to heterogeneous destinations. The data extraction for each resource is configured in the agent, through selectors. Selectors are key-value pairs, mapping a unique variable name, the key, with an expression to extract data elements, the value. Where keys are strings, values are specific to each data format. For example, with CSV data, values are column numbers, but with XML data, values are XPath query strings.

Delivery templates perform the transform and load process. Their configuration can have several variables, named within the *%{<variable name>}* expression. The custom template engine identifies variables at runtime that match the selector keys configured in the agent (being transferred in the event data). A variable in the template must have a corresponding selector in the agent. Yet, variables can be used multiple times throughout the template.

Additional features, such as data mapping in templates, can be achieved using a Ruby code script (with a mapping matrix, a switch statement or multiple condition tests) within the *${code(<ruby script>)}* function. We do not impose any restrictions to possible mappings, as long as the Ruby script is valid and outputs content suitable to the delivery format.

**Fig. 3:**
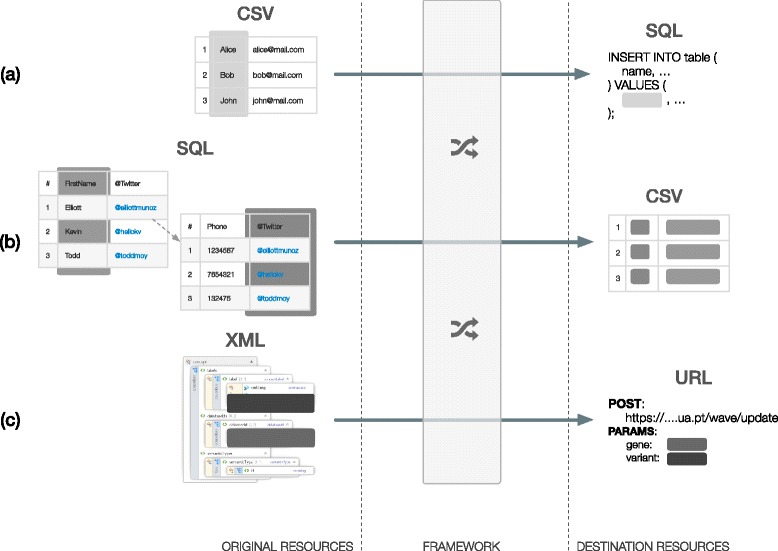
Applying data transformations. Data from Original Resources (in CSV/TSV, XML, JSON or SQL) can be easily translated and transformed (into URL requests, files, SQL queries or emails) using the framework’s templates: **a** CSV data are automatically inserted into a SQL database; **b** data are extracted from a SQL query into a CSV file; **c** XML elements are extracted (using XPath) and sent to a web service via POST request.

For instance, an integration task that extracts data from a CSV file to a SQL database has an agent with a set of selectors matching CSV column numbers, the selector value, with template variable names, the selector key. During the transform and load process, the Postman replaces SQL template variables (in the INSERT INTO… statement) with values obtained for each variable at each CSV row – Fig. 3.

Besides transforming static data for variables, templates can call internal functions or execute custom transformation code. Functions, named within *${<function name>}*, provide quick access to programmatic operations. For example, *${datetime}* outputs the time of delivery in the template.

As we cannot pre-emptively foresee all possible data mapping and transformation operations, we enable the execution of complex operations in Ruby code. The *${code(<ruby script>)}* function enables writing scripts that are evaluated during the delivery. This endows templates with a generic and flexible strategy for performing complex data deliveries. Taking this approach to the limit, we can perform a delivery that is entirely based on the execution of custom Ruby code. Code blocks can be of any size, as long as they are valid and finish returning a value. This covers both simple transformations such as numerical calculations, and complex operations such as matrix-based translations or mappings. Hence, we enable templates with conditional transformations, equations solving, strings manipulation, or calls to system functions, among many other operations. Further details are available in the framework documentation, online at https://bioinformatics.ua.pt/i2x/docs.

## Results

This framework brings a new perspective to the scientific data integration landscape, summarised in three main features, discussed in detail next.

- Automated real-time data integration is achieved through the deployment of intelligent agents, which can operate remotely, to monitor data sources.
- Improved data delivery to heterogeneous destinations using a template-based approach, allowing transmitting and transforming data.
- Advanced integration and interoperability, as we can use the framework to empower multiple service-oriented architectures, from publish/subscribe to cloud-based integration-as-a-service.

### Real-time content monitoring

Although the real-time paradigm is seldom applied to the life sciences domain, there are relevant research opportunities, besides genomics, open to exploration. For example, in health care, real-time analysis [28] can be used to improve patient data monitoring or, at an institutional level, to enhance the collection of statistical data [29].

This framework ensures real-time reliable data transmission and up-to-datedness. Real-time refers to the entire integration process. After the initial test, we heuristically decided that “every 5 minutes” represented the best trade-off between the overall application performance and researchers’ real-time demands to be the smallest update interval. Nevertheless, the platform can be setup to monitor data sources in any given interval, from every second to every year.

Local (server-side) or remote (client-side) agents perform resource-monitoring tasks. While server-side monitoring is enclosed within the server, remote monitoring brings three key benefits: distribution, improved load control and better security.

The ability to download, configure and execute monitoring tasks locally adds a unique distribution layer. At the architecture level, we can deploy and configure any number of remote agents, pushing data to the framework’s main server.

Local agent execution moves the monitoring schedule responsibility to the agent owners. As a result, agents’ scheduling is more flexible. Agents run as a standalone script with an associated configuration file. Scheduled tasks or manual ad-hoc execution (whenever data owners want to integrate/publish new data) automate the script execution.

At last, using client-side agents results in a more secure ecosystem. All communications with the platform are already secured through HTTPS. Yet, with client-side agents, sensitive content, such as authentication credentials or private API tokens, are not stored in the server. All configurations are saved locally, in a private JSON file. Moreover, user-based access tokens, unique 32 character strings, control the API data exchanges required for remote monitoring. Users can add one or more tokens and revoke existing ones in the server’s web interface. This token-based strategy also ensures that client-side agents only access the user's integrations and templates.

### Template-based delivery

The use of template-based engines in software is common and the integration domain is not an exception. From meta-programming [30] to service composition [31], templates are used to simplify recurring tasks and streamline processes.

Template-based integration strategies traditionally refer to the data extraction activity of the ETL warehousing workflow [32]. However, as this proposal uses intelligent agents for this task, templates' use fulfils the transform and load requirements. As detailed in the methods section, the framework includes a comprehensive template mechanism, based on variables and functions, to generate integration data for delivery to many destinations. This comprehensive approach is simple, yet flexible and powerful.

In summary, the framework allows four types of deliveries for now. These are succinctly described next.

- SQL queries to MySQL or Postures databases. As SQL is a powerful data manipulation language, complex query delivery can be combined with the agents’ detection output to perform advanced transformation.
- Send emails. Emails can be sent to multiple recipients (with CC and BCC also). All email properties (to, CC, BCC, subject and message) are available for customisation with the framework’s templating engine.
- File manipulation. We can define deliveries as writing files in the user platform workspace or in the user’s associated Dropbox account. We can create new files, or append or delete existing files. By managing files in the users’ Dropbox account, we ensure that they are always synchronised with external changes.
- Requests to URLs. The framework allows contacting miscellaneous services via HTTP GET or POST, enabling data exchanges in text, JSON or XML. This is the most powerful publishing method as it allows the interaction with most modern REST web services, which traditionally accept new data via POST and make data available via GET.

Currently, building tools to call REST services or manage files brings distinct requirements and implies custom *ad hoc* implementations. However, our template flexibility provides an abstraction on top of these methods. Whether we want to append lines to a CSV file, send an email or POST data to a REST service, users control the entire process in the server’s simplified interface.

### Advanced integration & interoperability

As previously mentioned, this proposal introduces an open source framework that can act as the foundation for distinct applications with distinct architectures. Whether we are dealing with event-driven applications or publish/subscribe environments, this framework can be easily adapted to support these systems.

#### Event-driven architecture

Traditional service-oriented architectures follow a request-response interaction model [33]. Although this basic operation principle sustains many systems, lack of support for responses to events is a major drawback [34].

Event-driven architectures adopt a message-based approach to decouple service providers from consumers [35]. In these service-oriented architectures, event detection is essential to get a reliable stream of changed data [36]. Event-driven architectures are used for direct responses to various events and for coordination with business process integration in ubiquitous scenarios.

Our framework enables this kind of reactive integration. Intelligent agents are configured to detect events, with the server acting as a message broker and router. For a truly event-driven environment, the framework also supports receiving events via push mechanisms. This means that we can deploy applications that fully operate on an event-driven paradigm.

#### Publish/subscribe

This framework is also an enabler of publish-subscribe software [37]. The basic principle behind this strategy revolves around a dynamic endpoint, the publisher, which transmits data to a dynamic set of subscribers [38]. Publish/subscribe architectures decouple communicating clients and complement event-driven architectures with the introduction of notifications. These can be a specialised form of events and ensure that we can actively push the desired data to subscribers in the shortest possible time.

Translating this basic principle to the framework’s model, we can see the agents as a controlled set of publishers (local or remote), which can be configured, using integrations, to interact with specific templates, the subscribers. This framework encloses the necessary tools and API that will allow applications to harvest the full potential of this paradigm in a seamless way.

Adopting the proposed work, data owners now have the tools to deliver custom notifications when new data are generated. This further advances the state of the art, namely on the life sciences [39], becoming vital when we consider the amount of legacy systems used to store data and the availability (or lack thereof) of interoperability tools to connect these systems.

Likewise, we can perform asynchronous push-based communication, broadcasting event data to any number of assorted destinations.

From an interoperability perspective, agents can also be on the subscriber end of the architecture. Events notification data can be pushed into to the system, triggering the integration in real-time.

#### Integration-as-a-service

Cloud-based integration-as-a-service is currently a major goal in service-oriented architectures [40]. Our framework empowers that paradigm, moving one step closer towards interoperable science data. This architectural approach abstracts algorithms, features, data or even full products as services [41], which should be available online via HTTP/S.

The framework can operate on both ends of the integration and interoperability spectrum. Besides being a service consumer, for data extraction and loading tasks, it also provides services for miscellaneous real-world problems.

As we embrace the Integration-as-a-Service notion, the relevance of frameworks to ease the process of exchanging and translating data amongst multiple service providers becomes clear. By combining the qualities of service-oriented, event-driven, publish/subscribe and cloud-based architectures, this framework endows users and developers with the required toolkit to build future-proof biomedical informatics software.

## Discussion

The proposed framework’s flexibility makes it suitable for miscellaneous integration use cases, connecting private or public data sources with existing services. More importantly, it allows researchers to create their own scenarios, with agents and templates suitable to their work.

The following discussion introduces a human variome data integration scenario and details some current limitations and future perspectives. This scenario can be tested at https://bioinformatics.ua.pt/i2x. This is a fully functional version of the platform, where everyone can register an account to create agents, templates and integrations.

### Automating variome data integration

Our first application scenario is provided in the configuration samples list available on the platform’s web interface. In it, we tackle a prime challenge for life sciences researcher regards the integration of human variome data: collecting unique mutations associated with a specific locus. This feature is already available in several systems. WAVe [42] or Cafe Variome [43] centralise data from distributed locus-specific databases (LSDBs) and make them available through web interfaces. The Leiden Open-source Variation Database (LOVD) provides a turnkey solution to launch new LSDBs, with web and database management interfaces [44]. These features make LOVD the *de facto* standard for LSDBs, with more than 2 million unique variants stored throughout 78 distinct installations. LOVD has an API enabled by default, which allows obtaining the full list of variants associated with a gene. These are returned in Atom format, a feed specification built in XML. In addition to LOVD, there are several other LSDBs using legacy formats, such as Excel or CSV files, or relational databases.

Despite the quality of available systems, data integration is limited by various constraints. For instance, the adopted pipeline - extract and curate data, enrich datasets, deliver results - creates a time-based snapshot of available human variome data. However, researchers require access to constantly updated datasets. To accomplish this we can use two distinct strategies: 1) we repeatedly launch our integration pipeline, processing everything from scratch or 2) we create an *ad hoc* integration solution tailored to this particular scenario. The maintenance and development effort underlying any of these strategies delays real progress.

**Fig. 4:**
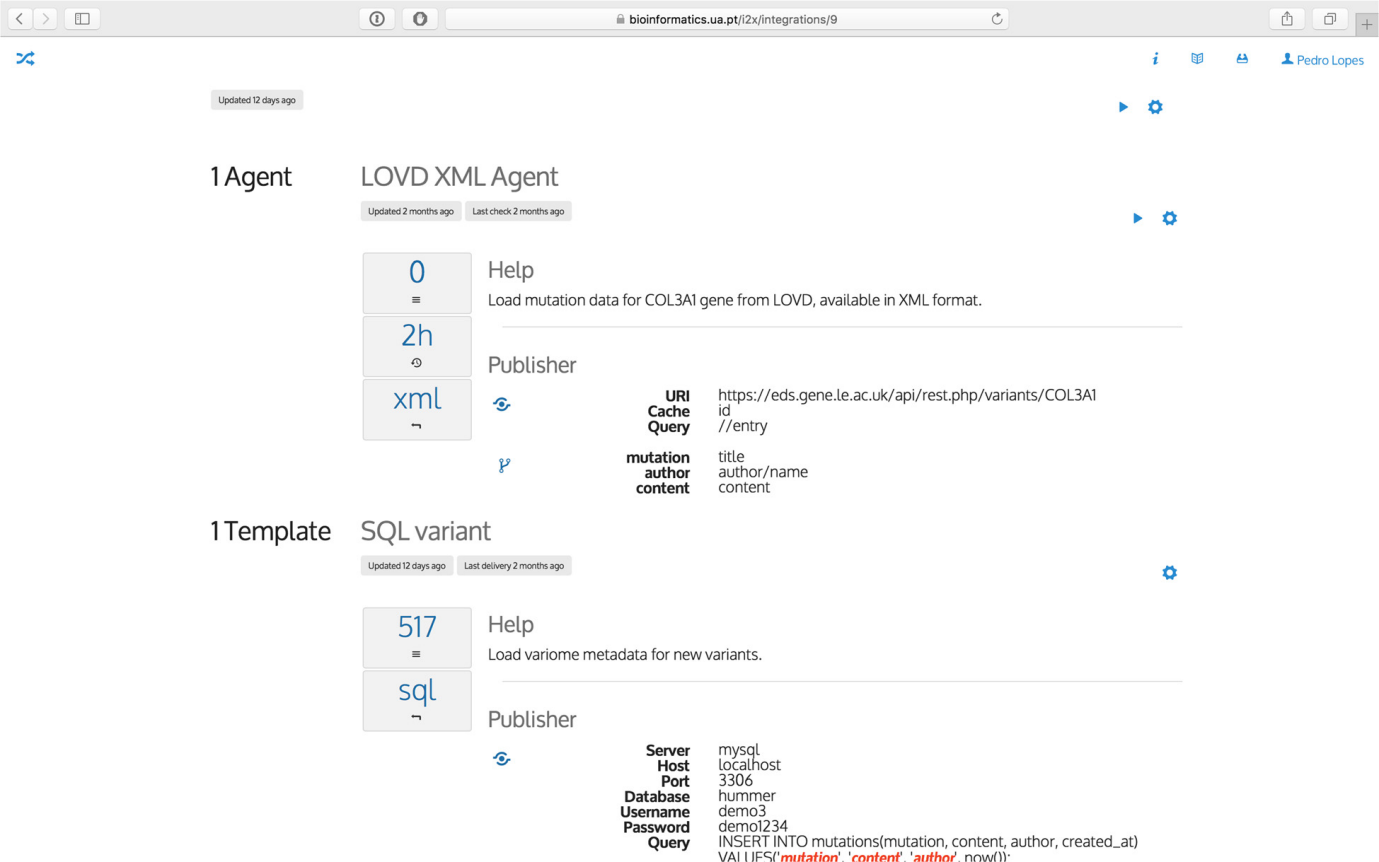
Web interface for proposed platform prototype. This interface highlights the integration configuration for automating human variome integration. This integration features one agent (LOVD XML Agent) and one template (SQL variant). The former configures how to extract mutation data from LOVD API and the latter specifies the configuration for storing extracted data in a relational database.

For simplicity purposes, we considered extracting data from the LOVD platform only. When curators submit new variants to LOVD, their data becomes available in the feed API. An agent is configured to monitor the feed for a single gene or, through the definition of a seed, for many genes based on a predefined list. After the initial data population process, events are detected when new variants are published. This starts a new integration task, which can deliver data directly to a database (using a SQL template) or send them to a URL-based service (using a URL route template). Figure 4 displays the platform prototype web interface showcasing the integration, agent and template configurations for this scenario.

This scenario is already in place in the data aggregation for WAVe’s backend (http://bioinformatics.ua.pt/wave/), which highlights three key benefits of the framework: autonomy, flexibility and data quality. First, the data warehousing process is triggered autonomously, without user intervention. Next, agents can track any number of genes and deliver data for new variants to any number of heterogeneous destinations. At last, variome datasets in the centralised application are always up-to-date with the latest discovered variants. Agent scheduling can monitor LSDBs frequently, resulting in improved database completeness.

Furthermore, in an ideal scenario, LSDBs owners can deploy local agents to monitor their databases internally or, using push, send events (with new variation data) directly to the main server platform for integration.

### Limitations and future perspectives

Although this open-source framework already supports advanced features for real-time content monitoring and data delivery, it has some limitations. Integration scenarios where the data acquisition involves combining data from multiple data sources or the execution of multiple data extraction steps require a more comprehensive integration solution. Likewise, there are several data compression and encryption methods that were not accounted for.

Hence, we are continually improving our solution, namely with the inclusion of new detection formats and delivery methods.

Data verification and rule processing are some of the main targets for future improvements. Our goal is to make a system that is completely independent from any origin or destination resource. This means that the platform should not rely on any external resources to ensure that data are new, which implies storing processed events in an internal database. As the platform needs to know what is new to trigger the integration process, we need to maintain a cache of everything the platform has already processed. These metadata could be complemented with delivery verification information, where we would store if and when the data were actually received by the destination resources. Although this would be another configuration burden for users, the overall integration algorithm will be improved with this feature.

We also plan to expand the framework’s data warehousing features by focusing on rule processing. The goal is to detach the ETL transform task from the delivery task, enabling more complex data transformation that obeys custom heuristics. In a simple use case, we want to perform the delivery only for data above a given threshold. Despite being able to perform these validations using Ruby code variables, simplifying these processes with dedicated methods will improve the framework’s usability.

In the long haul, we can further enhance rule processing with the inclusion of semantics. Latest developments on semantic web technologies and frameworks are responsible for an increased adoption within the life sciences field. As such, we plan to include support for LinkedData and SPARQL agents and delivery templates, and new inference and reasoning engines, allowing researchers to perform more complex operations with their data.

The mandatory configuration of scheduling properties for agents will also be the subject of future research work. We assume a constant flow of information in and out of the framework. In its current state, the framework is already tailored to scenarios where the data sources’ content change quite often. Traditionally, they require intensive manual effort to ensure the up-to-datedness of integrated data. With our proposal’s automation features and regular data monitoring, live data integration is ensured independently from the dataset update interval. For instance, regularly updated datasets can be monitored every 5 minutes or, in opposition, datasets that are seldom updated can be monitored daily or weekly.

To further improve this, we plan to include algorithms for the automatic identification of the best schedules for each resource. For instance, when a monitored resource generates events on a daily basis, the system should automatically understand that it is not efficient to schedule the resource for monitoring every 5 minutes. This will enhance the handling or large volumes of data and further improve the framework’s performance.

There are numerous high-quality data integration platforms. Nevertheless, it is common for existing solutions, such as Pentagon (http://www.pentaho.de/explore/pentaho-data-integration/) or Talend Open Studio (https://www.talend.com/products/talend-open-studio), among others, to have three major drawbacks: 1) they lack distribution, enforcing local access; 2) they operate in closed desktop environments; and 3) they lack automation features.

First, our approach enables the deployment of a distributed multi-agent architecture, where any number of agents can be running autonomously, processing data in any number of machines.

Moreover, monitoring agents can be deployed locally, where some desktop application to be integrated is running, or online, where most public systems are available. With this, we overcome traditional local-based integration problems. In our approach, a central web-based server operates online to control agents’ distributed execution and data delivery.

At last, current solutions require manual integration triggers, from script execution to loading cumbersome platforms, whereas our solution is fully automated, ensuring real-time integration.

## Conclusions

Nowadays, access to vast amounts of life sciences data is a commodity. Hence, modern integration strategies have arisen to better explore available information. In spite of their quality, existing models lack built-in mechanisms for handling change and time. That is, connecting data and services is a manual task, whose only results are time-limited snapshots.

With this research work we introduce a framework for enhanced integration and interoperability. This system’s innovative features - automation, real-time processing, flexibility and extensibility - convey true added value to biomedical data integration and interoperability.

Automation brings a new approach to the field, stimulating reactive and event-driven integration tasks. Furthermore, live data integration brought by automated and real-time intelligent agents ensure up-to-date information. The proposed framework is non-obtrusive, requires no changes in most original data sources, and can process data to and from heterogeneous data sources, making it an extremely flexible and adaptable framework.

This system is relevant for both researchers and developers: researchers can sign up to use the online platform and developers can download and modify the source code for local deployment. On the one hand, the platform’s streamlined configuration process puts a powerful integration and interoperability framework at the hands of less technical experienced users. We believe that the combination of the platform’s integration features with its easy-to-use web interface make the creation of integration tasks much more straightforward. Although the concept of integrations, agents and templates may be difficult to understand, once the user grasps these notions, deploying complex integration procedures becomes trivial. This enables anyone to create business-level data and service integration tasks with reduced effort. On the other hand, developers can download and use the framework open-source code. As the framework supports dynamic real-time message translation, formatting and delivery to multiple resources, it can play a central role in service-oriented architectures, from publish/subscribe to event-driven up to cloud-based integration-as-a-service environments.

This research work delivers a system that bridges the gap between data and services through a new intelligent integration and interoperability layer, further enabling the creation of next generation bioinformatics applications.

## Availability and requirements

**Project name:** i2x

**Project home page:**https://bioinformatics.ua.pt/i2x/

**Operating system(s):** Platform independent

**Programming language:** Ruby, JavaScript

**Other requirements:** Ruby web server, relational database management system

**License:** MIT

**Any restrictions to use by non-academics:** not applicable

## Abbreviations

API: Application programming interface
BCC: Blind carbon copy
CC: Carbon copy
CSV: Comma-separated values
ETL: Extract-transform-load
HTTP: Hypertext transfer protocol
HTTPS: HTTP secure
JSON: JavaScript object notation
LOVD: Leiden open-source variation database
LSDB: Locus-specific databases
MD5: Message digest algorithm 5
MIT: Massachusetts Institute of Technology
REST: REpresentational state transfer
SPARQL: SPARQL protocol and RDF query language
SQL: Structured query language
TSV: Tab-separated values
URI: Uniform resource identifier
URL: Uniform resource locator
XML: eXtensible Markup Language
